# Adenine crosses the biomarker bridge: from ‘omics to treatment in diabetic kidney disease

**DOI:** 10.1172/JCI174015

**Published:** 2023-10-16

**Authors:** Yelena Drexler, Alessia Fornoni

**Affiliations:** Katz Family Division of Nephrology and Hypertension, Department of Medicine, and Peggy and Harold Katz Family Drug Discovery Center, University of Miami Miller School of Medicine, Miami, Florida, USA.

## Abstract

Enabling the early detection and prevention of diabetic kidney damage has potential to substantially reduce the global burden of kidney failure. There is a critical need for identification of mechanistic biomarkers that can predict progression and serve as therapeutic targets. In this issue of the *JCI*, Sharma and colleagues used an integrated multiomics approach to identify the metabolite adenine as a noninvasive biomarker of progression in early diabetic kidney disease (DKD). The highest tertile of urine adenine/creatinine ratio (UAdCR) was associated with higher risk for end-stage kidney disease and mortality across independent cohorts, including participants with early DKD without macroalbuminuria. Spatial metabolomics, single-cell transcriptomics, and experimental studies localized adenine to regions of tubular pathology and implicated the mTOR pathway in adenine-mediated tissue fibrosis. Inhibition of endogenous adenine production was protective in a diabetic model. These findings exemplify the potential for multiomics to uncover mechanistic biomarkers and targeted therapies in DKD.

## Research gaps and opportunities in diabetic kidney disease

Diabetic kidney disease (DKD), defined broadly as kidney damage due to diabetes, is the leading global cause of progressive chronic kidney disease and end-stage kidney disease (ESKD) (1,2). DKD develops in approximately 40% of patients with diabetes, which has seen a remarkable increase in global prevalence and is expected to affect nearly 800 million people by the year 2045 (3). Despite recent advances in the therapeutic landscape, including the addition of sodium-glucose cotransporter-2 (SGLT2) inhibitors and nonsteroidal mineralocorticoid receptor antagonists to the armamentarium, patients with DKD continue to have a substantial residual risk of kidney and cardiovascular morbidity and mortality (4). The standard markers of estimated glomerular filtration rate (eGFR) and albuminuria can mislead clinicians in the initial stages of DKD, a time when structural damage is already occurring (5). eGFR can be normal or increased in the early stages of DKD, and the onset of macroalbuminuria may already coincide with advanced and irreversible lesions. In fact, the majority of patients with type 2 diabetes may have normoalbuminuria or microalbuminuria at the time that reduced kidney function is detected (5). The next frontier will involve the development of personalized therapies that target specific mechanisms of disease progression early in the disease course. A critical component of this effort is finding biomarkers that not only identify patients in the early stages of disease who are at high risk for DKD progression, but can also enable future drug development and clinical trials enriched for kidney outcomes. An approach based on individualized biomarker profiles will pave the way for precision medicine to change the trajectory of DKD for patients while they are still at an early stage of their disease when there is still a chance to intervene.

DKD provides a robust space for mechanistic biomarker discovery. Multiple interlinked processes contribute to the onset and progression of kidney damage in diabetes, including metabolic, hemodynamic, inflammatory, and fibrotic pathways (4). Several biomarkers of inflammation and fibrosis are independent predictors of DKD progression, including tumor necrosis factor receptor 1 (TNFR1), TNFR2, and kidney injury molecule-1 (KIM-1) (6, 7). TNFR1 and TNFR2 are circulating receptors of the proinflammatory cytokine TNF-α; KIM-1 is expressed in the apical membrane of proximal tubular cells in response to injury and promotes kidney fibrosis. Plasma levels of TNFR1, TNFR2, and KIM-1 are associated with higher risk of eGFR decline in patients with early or advanced DKD, and TNF receptors in particular have been associated with kidney outcomes, even among patients with diabetes and normoalbuminuria (6, 7). Notably, baseline levels of many of these inflammatory mediators are markedly intercorrelated, suggesting that these biomarkers are regulated by common upstream mechanisms that remain to be elucidated (8). Identification of such mechanistic biomarkers will require multimodal approaches, including the use of kidney biopsies for molecular analysis at the cellular level in combination with blood and urine markers. In this issue of the *JCI*, Sharma and colleagues used this groundbreaking approach to demonstrate that endogenous adenine drives kidney disease progression in DKD (9).

## Multiomics approach to elucidating mechanisms of kidney disease

**Figure 1:**
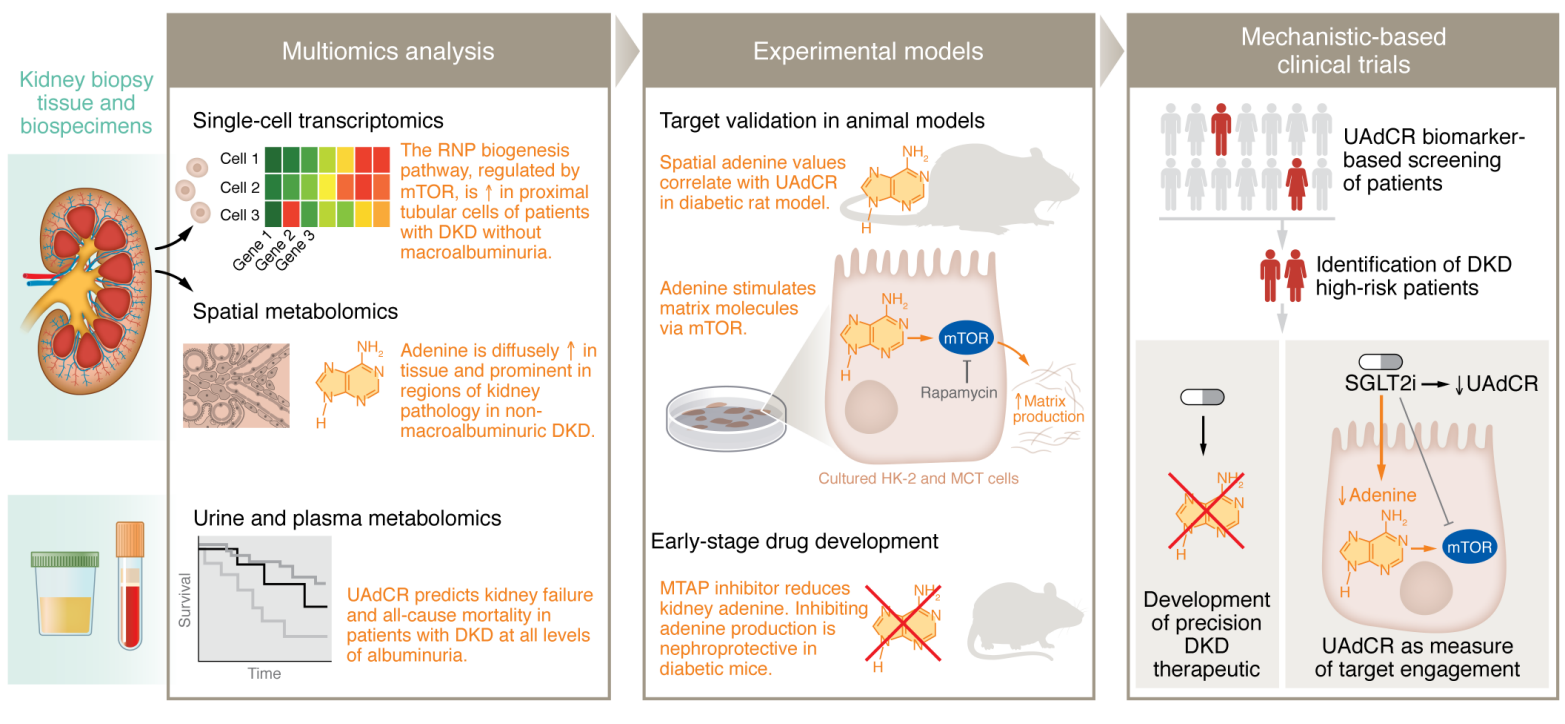
Multiomics approaches enable discovery of mechanistic biomarkers and targeted therapies in diabetic kidney disease. Kidney biopsy tissue and biospecimens (urine, plasma) are used to generate multiple types of molecular data, including single-cell transcriptomics, mass spectrometry imaging-based spatial metabolomics, and urine and plasma. These multimodal data are integrated using bioinformatic analysis to validate metabolites, cell types, and pathways. The pathways and biomarkers are then studied in experimental models to validate the target and allow for early-stage drug development. Finally, the novel biomarker is translated into mechanistic-based interventional clinical trials for clinical development of new DKD therapeutics. The UAdCR mechanistic biomarker could be used to stratify patients who are in the early stage of disease but at high risk for disease progression, to identify relevant subgroups of patients who are more likely to benefit from the targeted therapy for enrollment in clinical trials, and as a measure of target engagement in interventional trials of emerging therapeutics. DKD, diabetic kidney disease; HK-2, human kidney proximal tubular; MTAP, methylthioadenosine phosphorylase; MTC, murine kidney proximal tubular epithelial; mTOR, mammalian target of rapamycin; RNP, ribonucleoprotein; SGLT2i, sodium-glucose cotransporter-2 inhibitor; UAdCR, urine adenine/creatinine ratio.

Metabolomic analyses hold the potential to revolutionize the field of biomarker research through the identification and quantitation of all metabolites present in a given biofluid or tissue sample (10). Nontargeted metabolomics allows for global profiling of all possible metabolites and is typically used for discovery, while targeted metabolomics quantifies selected metabolites that are associated with a pathway of interest or disease state; it is typically hypothesis driven but can also serve as a method of discovery (11). As a next step, pathway enrichment analysis can help derive biological meaning from the metabolomic footprint. However, to truly understand disease processes and bridge the gap toward therapeutic development, it is imperative to consider the metabolomic footprint in the context of the structure-function relationship at the level of the diseased tissue. At the forefront of this effort, the Kidney Precision Medicine Project (KPMP) study has developed a human kidney tissue atlas — using kidney biopsies from unaffected patients and those with kidney disease — that makes it possible to map structure-function relationships at the single-cell level and understand how cell-type-specific processes and their metabolites are altered in different disease states (12). The data derived from this effort are extremely robust and diverse, including transcriptomics using single-cell and single-nucleus RNA-sequencing technologies, regional (i.e., glomerular and tubulointerstitial) bulk transcriptomics, proteomics, metabolomics, and mass spectrometry imaging–based spatial metabolomics. The kidney is uniquely suited to this effort, with its diversity of cellular subtypes — including glomerular, proximal tubular, vascular, and resident and circulating immune cells — and spatial organization. Integration of these multiomics data sets provides a crucial framework for understanding molecular mechanisms of disease and for identifying potential predictive biomarkers that are then validated in animal models, investigated in patient cohorts for linking to clinical phenotypes, and ultimately mapped in individual patients (Figure 1).

## Endogenous adenine in diabetic kidney disease progression

In this issue of the *JCI*, Sharma and colleagues used an integrated multiomics approach to demonstrate that endogenous adenine mediated injury in DKD models and predicted the progression of DKD in patients (9). In a previous analysis, the investigators used a high-throughput untargeted metabolomic platform combined with machine learning to examine nearly 700 metabolite ions in the urine of participants from the Chronic Renal Insufficiency Cohort (CRIC) study and identified 99 metabolites that were associated with progression to ESKD after adjusting for clinical variables (13). Pathway analysis revealed metabolic pathways that were consistently enriched, and a targeted assay validated 13 of 15 metabolites — including adenine — that were associated with ESKD. Adenine is a purine nucleobase that is known to induce progressive kidney damage, including glomerular and proximal tubular injury and induction of proinflammatory and profibrotic pathways, as an exogenous toxin in animal models (14). Adenine also stimulates the mTOR complex 1 (mTORC1), which plays a key role in promoting ribosome biogenesis — an energy-intensive and highly regulated cellular process — and enhancing global protein synthesis and cell growth (15). While exogenous adenine is a known mediator of injury in kidney disease models, much less is known about the role of endogenous adenine in the initiation and progression of kidney disease.

In a multifaceted approach, Sharma and colleagues first used urine metabolomics to determine whether urine adenine levels can predict kidney failure and mortality, even among patients with normal or elevated GFR without macroalbuminuria. Specifically, the authors evaluated several independent, diverse cohorts of patients with diabetes for an association between baseline urine adenine/creatinine ratio (UAdCR) and clinical endpoints — namely ESKD and all-cause mortality (9). They determined urine levels of adenine normalized to urine creatinine using a rapid throughput assay that combines a microfluidic chip for metabolite separation with mass spectrometry. In a sample of over 900 participants with diabetes and reduced eGFR from the CRIC study, the highest UAdCR tertile compared with the lowest was associated with a 1.57-fold higher risk for ESKD or all-cause mortality (adjusted HR, 1.57; 95%, CI 1.18–2.10) independent of baseline eGFR and albuminuria. Similar robust associations between UAdCR and risk of ESKD were found when analyzing cohorts without macroalbuminuria. Among 551 CRIC participants with reduced eGFR and without macroalbuminuria, the highest UAdCR tertile compared with the lowest identified participants with a 2.36-fold higher risk for ESKD (adjusted HR, 2.36; 95%, CI 1.26–4.39). Findings in CRIC were validated in separate cohorts of patients with diabetes without macroalbuminuria, including American Indians with preserved measured GFR and Southeast Asian participants with reduced eGFR. Second, the investigators evaluated the effect of different glycemic conditions and therapeutic intervention with the SGLT2 inhibitor empagliflozin on UAdCR in nonmacroalbuminuric participants. Despite no effect of acute hyperglycemia on UAdCR, eight weeks of treatment with empagliflozin reduced UAdCR levels by more than one-third, suggesting that UAdCR could be used to monitor response to treatment in nonmacroalbuminuric patients. Third, the investigators used spatial metabolomics to determine the regional localization of adenine in kidney biopsy tissue sections. While adenine was present at low intensity in healthy control kidney, adenine was increased overall and in areas of glomerular, tubular, and vascular pathology among patients with diabetes, even among those without macroalbuminuria. Fourth, the investigators used single-cell transcriptomics to analyze differentially expressed genes from proximal tubular cells in patients with DKD compared with those in individuals used as healthy controls. Pathway enrichment analysis identified the ribonucleoprotein biogenesis pathway as the top upregulated pathway in DKD biopsies from the KPMP and a second independent study. Additional experiments provided further mechanistic insights. Adenine stimulated matrix molecules in proximal tubular cells, an effect mediated by the mTOR pathway and blocked via inhibition of mTORC1 with rapamycin. Adenine administration to healthy mice increased levels of the injury markers soluble TNFR1 (sTNFR1) and KIM-1 and induced kidney hypertrophy, kidney mTOR activity, and kidney matrix production. Finally, blocking endogenous adenine production using a specific small-molecule inhibitor of methylthioadenosine phosphorylase was protective in diabetic mice, as evidenced by improvements in diabetic kidney hypertrophy, kidney function, and injury biomarkers, including kidney KIM-1 levels.

## Clinical and research implications

Sharma and colleagues have demonstrated how an integrated multiomics approach can bridge the gap between identifying mechanistic biomarkers and developing therapeutics based on those targetable pathways. This approach also provides a framework to elucidate mechanisms of kidney protection for known therapeutics and to promote drug discovery. SGLT2 inhibitor treatment results in dose-dependent improvements in measures of kidney function and inflammatory and oxidative stress in animal models of adenine-induced chronic kidney disease (16, 17) and leads to early decreases in kidney injury markers independently of albuminuria in individuals with diabetes. Furthermore, there is recent evidence that SGLT2 inhibitors restore diabetes-induced metabolic perturbations via suppression of mTORC1 signaling in proximal tubular cells in young individuals with type 2 diabetes and in a diabetes mouse model (19). The study by Sharma and colleagues suggests that some of the benefit of SGLT2 inhibitors may derive from reduced endogenous adenine levels, as measured by UAdCR. SGLT2 inhibitors may in fact mitigate adenine-induced tubular injury via the mTOR pathway as a cellular mechanism of kidney protection. Importantly, inhibition of endogenous adenine production with the specific small-molecule inhibitor MTDIA, which reduced kidney adenine and appears to be well tolerated in mice, can be investigated as a potential therapeutic, using the UAdCR biomarker as a measure of target engagement (9, 20).

With this breakthrough study by Sharma and colleagues, we can more clearly envision a future where multiomics approaches enable discovery of predictive biomarkers — such as the UAdCR — that can be assayed routinely and noninvasively early in the disease course. This example should serve as a call to accelerate drug discovery, which will be needed to truly revolutionize our ability to prevent DKD.

## Version 1 10/16/2023

Electronic publication
